# Mechanisms and current advances in treating *KRAS*-mutated lung cancer

**DOI:** 10.1016/j.pccm.2025.08.001

**Published:** 2025-09-16

**Authors:** Cynthia Hsin-Ya Chao, Yuanpu Peter Di

**Affiliations:** Department of Environmental and Occupational Health, University of Pittsburgh, Pittsburgh, PA 15260, USA

**Keywords:** Lung cancer, Non-small cell lung carcinoma, Resistance, *KRAS*, Targeted therapy

## Abstract

Lung cancer is the leading cause of cancer-related deaths, with the highest mortality among all cancers. Despite the significant advances in cancer treatments in recent years, especially with the development of checkpoint inhibitor immunotherapy, a definitive treatment has yet to be discovered to cure lung cancer. Lung tumorigenesis involves genetic alterations in a multi-step process with heterogeneity and diversity, such that even though cigarette smoke has been widely acknowledged as a major associated risk factor, many non-smokers still develop lung cancer. Among lung cancers, 85 % are non-small cell lung carcinoma (NSCLC), with adenocarcinoma as the most prevalent NSCLC subtype, making up around 40 % of all lung cancers. The major genetic mutation drivers include the epidermal growth factor receptor (*EGFR*), anaplastic lymphoma kinase (*ALK*), and Kirsten rat sarcoma viral oncogene homolog (*KRAS*). While the advancement of newer-generation anti-cancer drugs has successfully treated *EGFR*- and *ALK*-associated lung cancers, *KRAS* mutation-associated lung cancer remains extremely challenging and has limited therapeutic options. This review outlines the etiology, epidemiology, and categorization of lung cancer, describing the current therapeutic options and limitations, with a focus on the most challenging-to-treat *KRAS*-mutated lung cancer. Furthermore, this paper highlights the current state and development of *KRAS*-mutated cancer treatment by describing the mechanisms and utilities of various *KRAS*-targeted therapies entering clinical trials, and it underlines the most promising treatment options.

## Introduction

As the most diagnosed cancer worldwide, lung cancer remains the leading cause of cancer-related deaths overall, accouting for almost one in five total cancer deaths.^1^^,^^2^ The latest estimates showed 2.5 million new cases and 1.8 million deaths in the year 2022 alone.^1^

Based on the histological types, lung cancer can be mainly classified into small cell lung cancer (SCLC) and non-small cell lung cancer (NSCLC). The former accounts for 15 % of all lung cancer, while the latter accounts for about 85 %. NSCLC can be further subdivided into three main subtypes: adenocarcinoma (40 %), squamous cell carcinoma (25–30 %), and large cell carcinoma (5–10 %).^3^ Targetable molecular mutations can be found in approximately 60 % of lung adenocarcinomas in the Western population and 80 % in the Asian population.^4^ Within lung adenocarcinoma, the three most common genetic drivers are epidermal growth factor receptor (*EGFR*), anaplastic lymphoma kinase (*ALK*), and Kirsten rat sarcoma viral oncogene homolog (*KRAS*). The activation of these mutations usually occurs in a mutually exclusive manner, creating distinct subgroups of the disease. While targeted therapies were developed for *EGFR*- and *ALK*-mutated lung cancers, *KRAS*-mutated lung cancer remained undruggable for decades until recent breakthroughs.

## Epidemiology of *KRAS*-associated lung cancer

**Fig. 1:**
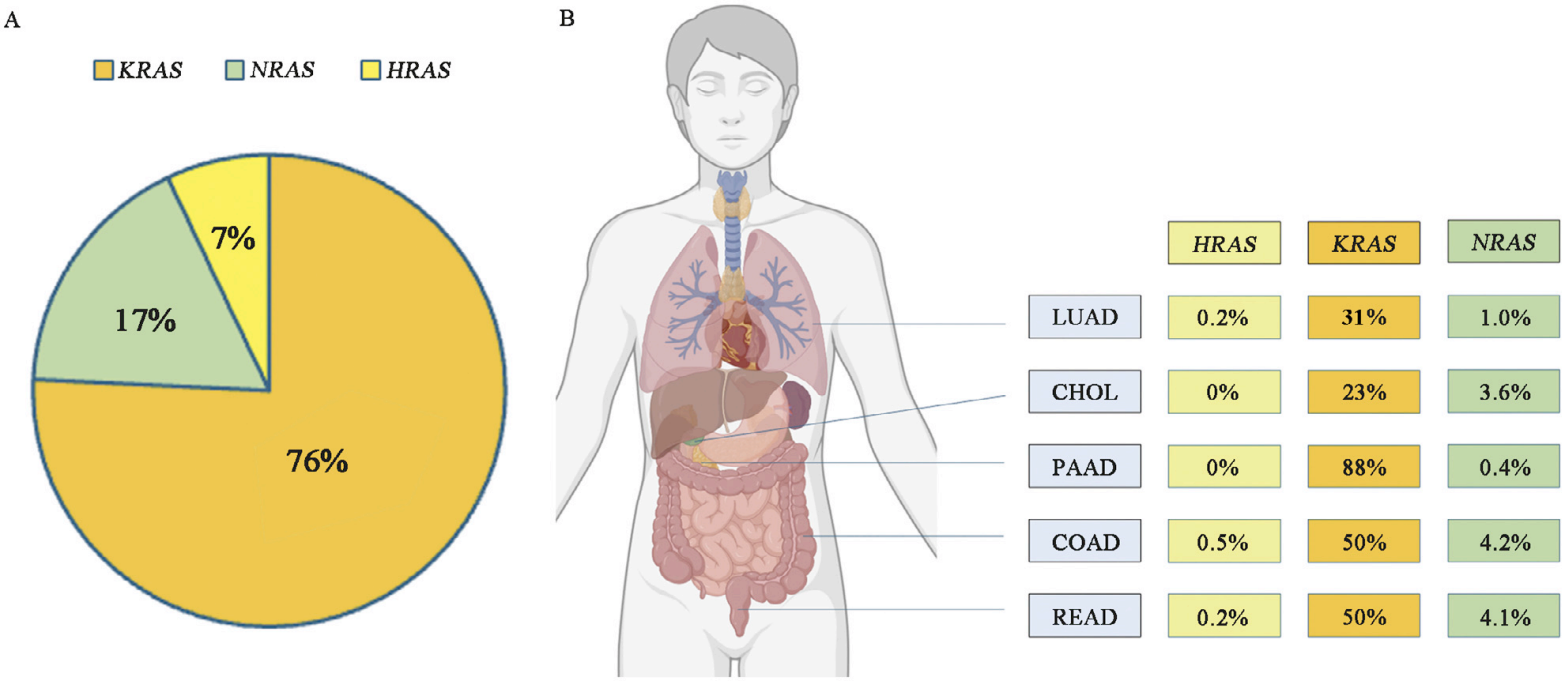
(A) Mutation frequency of *RAS* isoforms in cancer. *KRAS* is the most frequently mutated of the three Ras isoforms. (B) Prevalence of HRAS, *KRAS*, and *NRAS* across common cancer types. *KRAS* remains the most prevalent isoform across listed cancers, with 31 % of lung cancer harboring *KRAS* mutation. CHOL: Cholangiocarcinoma; COAD: Colon adenocarcinoma; *HRAS*: Harvey rat sarcoma viral oncogene homolog; *KRAS:* Kirsten rat sarcoma viral oncogene homolog; LUAD: Lung adenocarcinoma; *NRAS*: Neuroblastoma rat sarcoma viral oncogene homolog; PAAD: Pancreatic adenocarcinoma; READ: Rectal adenocarcinoma.

Four decades ago, rat sarcoma (RAS) genes were the first oncogenes identified in the field of cancer research^5^; yet, their seniority in discovery has not led to a breakthrough in treatment, as no drug was perceived to be effective against the *RAS* mutation. Studies indicate that approximately 19 % of cancer patients encompass *RAS* mutations, meaning about 3.4 million new cases diagnosed yearly worldwide require anti-RAS therapies.^6^ Among the RAS GTPase family members, three isoforms have been identified: Harvey rat sarcoma viral oncogene homolog (HRAS), neuroblastoma rat sarcoma viral oncogene homolog (NRAS), and *KRAS*. While these *RAS* oncogenes share similar molecular structures, they exhibit distinct expression patterns that contribute to their varying prevalences across human cancers. Among the *RAS* oncogenes, *KRAS* is the most frequently mutated and is responsible for about ∼76 % of *RAS*-mutated cancers, while *NRAS* accounts for 17 % and *HRAS* for 7 % (Fig. 1). *KRAS* mutations are especially prevalent in cancers such as pancreatic adenocarcinoma (88 %), lung adenocarcinoma (31 %), and colorectal cancers (45–50 %). In contrast, *NRAS* mutations are most associated with melanomas, acute myeloid leukemia, and thyroid carcinoma, whereas *HRAS* mutations are most often seen in pheochromocytoma and paraganglioma, thymoma, and head and neck squamous cell carcinoma.^6^

## *KRAS* mutation subtypes and clinical characteristics

In Western countries, *KRAS* is the most frequently mutated genetic driver in lung cancer, accounting for approximately 26.1 % of lung adenocarcinomas. In contrast, the prevalence of *KRAS* mutations in Asian populations is lower, ranging from 10 % to 15 %, surpassed by *EGFR* mutations.^7^ A large Chinese cohort study similarly reported *KRAS* as the second most common driver mutation, present in approximately 10 % of NSCLC patients. Chinese patients with *KRAS* mutation had a shorter median overall survival than wild-type tumors (15.1 *vs.* 26.7 months).^8^

Oncogenic *KRAS* mutation subtypes are primarily classified based on the type of nucleotide substitutions: transversion mutations (G→T or G→C), such as G12C, G12V, G12A, and G12R, and transition mutations (G→A), such as G12D, G12S, and G13D. Over 80 % of the *KRAS* mutations occur at codon 12, where the glycine amino acid is substituted, most commonly by cysteine (G12C), followed by valine (G12V) and aspartic acid (G12D).^9^ The distribution of these mutation subtypes also varies significantly across different cancer types, reflecting the complex and heterogeneous nature of *KRAS*-driven cancers. *KRAS* glycine-to-cysteine (G12C) mutation accounts for approximately 13 % of all lung adenocarcinoma patients, while *KRAS* glycine-to-aspartic acid (G12D) mutation is found in 4 % of lung adenocarcinoma patients.^9^

Clinically, *KRAS*-mutated lung cancer is associated with older age, male gender, and smoking history. A meta-analysis demonstrated that never-smokers have significantly reduced odds of harboring *KRAS* mutations compared to ever-smokers, with odds ratios of 0.22 in Caucasians and 0.39 in Asians.^10^ Transversion mutations (e.g., G→C; also known as G12C) were more common in ever-smokers, while transition mutations (G→A; also known as *KRAS* G12D) were more commonly seen in never-smokers.^11^ Demographic patterns show *KRAS* G12C mutations are more frequent in white females, although they are more common in males among Asians.^12^ Among races, the frequency of *KRAS* G12C in NSCLC patients of Caucasian and African American ethnicity is 13 % and 10.9 %, respectively, compared to Asians (3.6–4 %).^12^^,^^13^ However, this estimate may not fully reflect intra-Asian variability. A multicenter Chinese study reported that ∼10 % of Chinese NSCLC patients harbor *KRAS* mutations, with 30 % of these being *KRAS* G12C, equating to approximately 2.9 % of Chinese NSCLC cases. The *KRAS* G12C subtype was also more frequently observed in Chinese male smokers.^8^ This discrepancy may be attributed to differences in study population, geographic variation, detection method, and environmental and lifestyle factors. For instance, vegetable intake has been shown to have a negative correlation with the frequency of *KRAS* transition mutations.^14^ The molecular landscape of *KRAS*-mutated lung cancer also includes concurrent genetic alterations. Among tumors with *KRAS* transversion mutations, tumor protein 53 (*TP53*) and *PIK3CA* co-mutations are observed in 24.6 % and 13.1 % of cases, respectively. In contrast, *PIK3CA* and *TP53* mutations are found in 21.1 % and 15.8 % of tumors with transition-type *KRAS* mutations.^14^ Previously, it was thought that *KRAS* and *EGFR* were mutually exclusive, but much emerging evidence suggests that rare co-occurrences exist, potentially due to acquired resistance or *de novo* mutational events.^14^

Moreover, certain subtypes portend unfavorable outcomes. G12C and G12V point mutations are associated with the worst survival compared to other *KRAS* mutation subtypes.^15^ *KRAS* mutation with serine/threonine kinase 11 (*STK11*) tumor suppressor gene loss had particularly dismal outcomes, with a median overall survival of only 0.9 years and a tendency to present at a younger age (median 61 years).^11^

## Molecular mechanisms of *KRAS*

As the most frequently mutated oncogene family in cancer, RAS proteins function as a cellular signaling network regulatory switch that controls cell proliferation and differentiation. RAS is a guanine nucleotide-binding protein that becomes activated when bound to guanosine triphosphate (GTP) but is inactivated when bound to guanosine diphosphate (GDP). To terminate RAS activity, RAS needs to interact with a GTPase-activating protein to stimulate the GTPase protein, converting GTP back to GDP. Cancer-causing mutations in *RAS* are thought to be related to the state of GTP-bound RAS that led to the propagation of a cascade of downstream effector pathways resulting in unstoppable carcinogenesis (Table 1).

**Table 1:** Main components of the RAS signaling pathway.

Key players	Role	Function
RTK	Receptor	Initiates signal transduction
SOS	Component of the GEF, a direct activator of RAS	Promotes the exchange of inactive GDP for active GTP form
SHP2	Component of the GEF, upstream modulator	Helps activate RTK and recruit SOS
GRB2	Component of the GEF	Links activated RTKs to SOS
GEF	Activator	Enables KRAS to switch to its active GTP-bound state, triggering downstream signaling
GAP	Inactivator	Accelerates GTP hydrolysis, switching it to inactive GDP-bound form
RAF	Component of the MAPK pathway	Phosphorylates and activates MEK, initiating MAPK cascade
MEK	Dual-specificity kinase; component of the MAPK pathway	Phosphorylates ERK, propagating signal downstream
ERK	Effector kinase; component of the MAPK pathway	Terminal kinase in MAPK cascade
PI3K	Lipid kinase/signal transducer; component of the PI3K/AKT pathway	Converts PIP2 → PIP3, recruiting AKT to membrane
AKT	Effector kinase; component of the PI3K/AKT pathway	Promotes cell survival, growth, and metabolism
mTOR	Central growth regulator; component of the PI3K/AKT pathway	Integrates growth signals to promote protein synthesis and inhibit autophagy
Cyclin D1	Cell cycle activator	Binds to CDK4/6 to drive G1 phase to S phase
CDK4 and CDK6	Cell cycle drivers	Phosphorylates Rb protein to release E2F to enable entry into the S phase

AKT: Protein kinase B; CDK4/6: Cyclin-dependent kinase 4/6; E2F: E2F transcription factor; ERK: Extracellular signal-regulated protein kinase; GAP: GTPase activating protein; GDP: Guanosine diphosphate; GEF: Guanosine nucleotide exchange factor; GRB2: Growth factor receptor-bound protein 2; GTP: Guanosine triphosphate; KRAS: Kirsten rat sarcoma viral oncogene homolog; MAPK: Mitogen-activated protein kinase; MEK: Mitogen-activated protein kinase kinase; mTOR: Mammalian target of rapamycin; PI3K: Phosphoinositide-3-kinase; PIP2: Phosphatidylinositol 4,5-bisphosphate; PIP3: Phosphatidylinositol (3,4,5)-trisphosphate; RAF: Rapidly accelerated fibrosarcoma; RAS: Rat sarcoma; RTK: Receptor tyrosine kinase; SHP2: Src homology region 2 domain-containing phosphatase-2; SOS: Son of sevenless.

When *KRAS* receives a signal from receptor tyrosine kinase (RTK) on the cell surface, it is converted from a GDP-bound inactive form to a GTP-bound active form by guanosine nucleotide exchange factors (GEF), which include son of sevenless (SOS), SRC homology region 2 domain-containing phosphatase-2 (SHP2), and growth factor receptor-bound protein 2 (GRB2). Extracellular signal-regulated GTPase activating protein converts the GTP-bound active form back to GDP-bound inactivate form.^16^

GTP-bound *KRAS* activates a few downstream signaling pathways, including the mitogen-activated protein kinase (MAPK) and phosphoinositide-3-kinase (PI3K). Although the MAPK and PI3K pathways both play crucial roles in cell signaling, they have distinct functions. The MAPK pathway primarily influences proliferation, differentiation, and survival, while the PI3K pathway regulates anabolic processes such as protein synthesis, metabolism, and angiogenesis.

The MAPK pathway consists of rapidly accelerated fibrosarcoma (RAF) kinases, MAPK kinase (MEK),^17^ and extracellular signal-regulated protein kinase (ERK). When RAS is activated, its conformational change allows it to bind and phosphorylate the RAF enzyme. The activation of RAF will then activate MEK, initiating the MAPK cascade, also known as the RAS-RAF-MEK-ERK signal transduction cascade. MEK acts as a dual-specificity kinase that can phosphorylate both tyrosine and threonine residues in its target proteins, specifically ERK. Once ERK is activated, it translocates to the nucleus to modulate gene expression in cell division and survival.

Parallel to this, the activation of the PI3K pathway leads to the production of phosphatidylinositol (3,4,5)-triphosphate (PIP3), which recruits and activates the downstream effector, protein kinase B (AKT). Activated AKT then subsequently activates the mammalian target of rapamycin (mTOR), resulting in the transcription of genes needed for protein synthesis, cell proliferation, and survival.

RALA and RALB are RAS downstream small GTPases, and mutated KRAS can lead to tumorigenesis by activating RALA and RALB, which regulate cell proliferation, invasion, and metastasis. Once signal transduction reaches the nucleus, cyclin-dependent kinases (CDK4 and CDK6) form complexes with D-type cyclins and phosphorylate retinoblastoma protein (RB) to release early region 2 binding factor (E2F) transcription factor, driving the transition from G1 to the S phase of the cell cycle.

Evidence has shown that different *KRAS* isoforms have various degrees of dependence on these major downstream pathways. *KRAS*-G12D mutations have a preference for PI3K/AKT/mTOR pathway,^15^ while G12C and G12V prefer RAS-related protein (RALA/B), and G12A, G13D, and Q61D favor the MAPK pathway.^17^

## Impacts of *KRAS* on current treatments

Early efforts to combat *RAS* mutations primarily focused on *HRAS* because it was discovered first, but results could not be translated clinically. Despite the molecular similarity between *HRAS* and *KRAS*, treatments for *HRAS* do not successfully apply to *KRAS. KRAS*-mutated tumors are among the most aggressive malignancies and refractory to treatment.

*KRAS* mutants represent approximately 27.5 % of NSCLC patients, while *KRAS* wild-type represents 72.5 % of NSCLC patients.^18^ *KRAS*-mutant cancers carry a driver mutation within the *KRAS* gene. In contrast, *KRAS* wild-type cancers have a normal, non-mutated *KRAS* allele but may still harbor mutations in other oncogenes or tumor suppressors. Consequently, patients with wild-type *KRAS* are not candidates for *KRAS*-directed targeted therapies but may benefit from immunotherapy, chemotherapy, and other targeted therapies for other driver genes. Wild-type *KRAS* alleles, specifically *KRAS*m/WT+, are less sensitive to inhibitors of the downstream *KRAS* pathway.^19^

Targeted treatments on NSCLC with actionable drivers such as *EGFR* and *ALK* have shown high clinical efficacy compared with those without molecular targets. However, the three most common driver mutations, *KRAS, EGFR*, and *ALK*, are frequently mutually exclusive.^20^ Even though the KRAS-MAPK pathway is downstream of *EGFR* signaling, *KRAS*-mutated cancers do not respond to *EGFR* tyrosine kinase inhibitors (TKIs). Furthermore, *KRAS* activation often triggers downstream pathways of *EGFR* signaling while bypassing the need for *EGFR* itself.

The treatment algorithm for *KRAS* G12C-mutated NSCLC follows the same ESMO (European Society for Medical Oncology Clinical Practice Guidelines) guideline for non-oncogene-addicted metastatic NSCLC.^21^ The treatment strategy is dependent on the Eastern Cooperative Oncology Group (ECOG) performance status (PS) and programmed death ligand 1 (PD-L1) expression level. Cancer patients with good performance status (PS 0–1) can be initiated with immunotherapy combined with platinum-based chemotherapy. Those with an intermediate performance status (PS 2) and PD-L1 expression of 50 % or higher may use a single-agent immune checkpoint inhibitor; however, if PD-L1 expression is <50 %, combination platinum-based chemotherapy is preferred. Finally, patients with NSCLC and poor performance status (PS 3–4), regardless of PD-L1 status, are given supportive care. Systemic therapy should be offered to patients with stage IV oligometastatic NSCLC^22^ (Table 2).

**Table 2:** Summarized treatment guideline of stage IV *KRAS*-mutated NSCLC.

PD-L1 expression	Condition	First line	Second line
Any	Oligometastatic	Systemic + LRT	Continue systemic therapy
	PS 0–1	ICI+ PDC	Sotorasib/adagrasib (if *KRAS* G12C-mutated)
	PS 3–4	Best supportive care	Best supportive care
PD-L1 <50 %	PS 2	PDC	ICI
PD-L1 ≥50 %	PS 0–2	ICI	PDC

ICI: Immune checkpoint inhibitor; *KRAS:* Kirsten rat sarcoma viral oncogene homolog; LRT: Local regional therapy; NSCLC: Non-small cell lung carcinoma; PDC: Platinum-doublet chemotherapy; PD-L1: Programmed-death ligand 1; PS: Eastern Cooperative Oncology Group performance status.

The use of single-agent ICI has become the standard treatment for patients with squamous-cell lung carcinoma as well as non-squamous non-small-cell lung carcinoma and a high PD-L1 expression.^22^ However, the response has been poor, and resistance to ICIs is common in *KRAS* mutated lung cancers because oncogenic KRAS signaling can repress critical immune regulatory proteins, such as components of the MHC-I antigen presentation pathway. The TAILOR trial confirmed that the presence of *KRAS* mutations also has a negative prognosis on NSCLC treated with first-line platinum-containing chemotherapy (PDC).^23^ The median overall survival (OS) found in the trial showed that patients lived for 14.3 months in wild-type *KRAS*, while only 10.6 months in mutated *KRAS* patients. A Chinese retrospective study showed that the progression-free survival (PFS) for chemotherapy as a first-line treatment was only 4 months.^24^

Different *KRAS* mutations have different clinical responses and overall survival to immune checkpoint inhibitors.^25^ Among patients with *KRAS*-mutated NSCLC harboring G12C variants, those with higher tumor mutation burden and PD-L1 expression ≥50 % might demonstrate greater sensitivity to immune checkpoint inhibitors.^13^ Among *KRAS*-mutant patients who received immune checkpoint inhibitor therapy, the PFS in those harboring G12C (3.4 months) was similar to that with G12V (4.2 months), but significantly longer than that with G12D mutation (2.0 months).^14^ In *KRAS*-mutant cancers, concomitant mutations critically influence immunotherapy efficacy. *STK11* and Kelch-like ECH-associated protein 1 (*KEAP1*) co-mutations correlated with poor immunotherapy outcomes in patients with *KRAS* mutation.^26^ In contrast, patients with *KRAS*/TP53 co-mutations respond better to immunotherapy (objective response rate [ORR]: 35.7 %) compared to those with *KRAS*/STK11 co-mutations (ORR: 7.4 %).^27^

## Development of targetable drugs

**Fig. 2:**
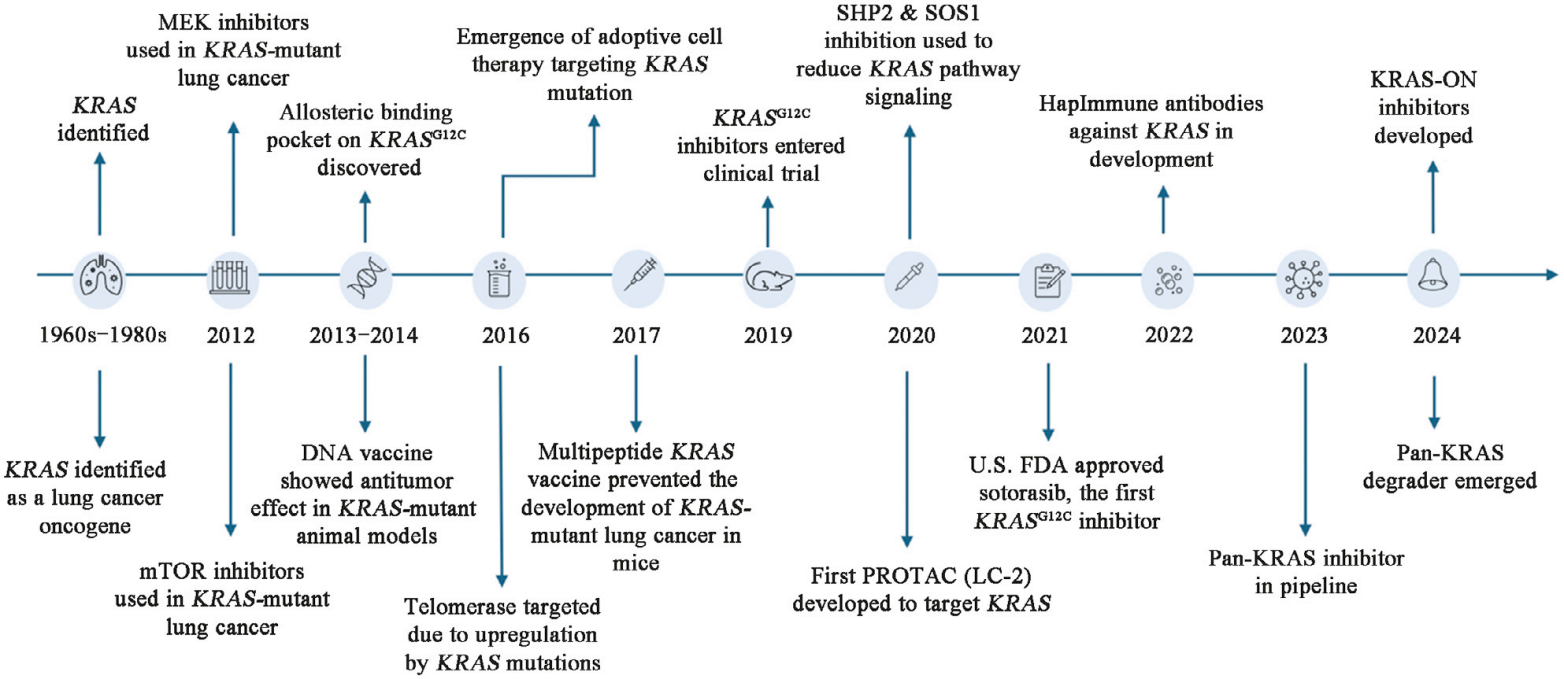
Timeline of advancements in *KRAS*-targeted therapies. FDA: U.S. Food and Drug Administration; G12C: Glycine-to-cysteine substitution at codon 12 of *KRAS*; G12O: Glycine-to-other amino acid substitution at codon 12 of *KRAS; KRAS*: Kirsten rat sarcoma viral oncogene homolog; LC-2: First PROTAC degrader targeting *KRAS*; MAPK: Mitogen-activated protein kinase; MEK: Mitogen-activated protein kinase kinase; mTOR: Mammalian target of rapamycin; PI3K: Phosphoinositide 3-kinase; PROTAC: Proteolysis-targeting chimera; SHP2: SH2 domain-containing phosphatase 2; SOS1: Son of sevenless homolog 1.

For decades, lung cancer with *KRAS* mutations did not have a direct inhibitor as it had been deemed undruggable due to the structural characteristics of the target domain (Fig. 2, Supplementary Table 1^28^, ^29^, ^30^, ^31^, ^32^, ^33^, ^34^, ^35^, ^36^, ^37^, ^38^, ^39^, ^40^, ^41^, ^42^, ^43^, ^44^, ^45^^–^ ^46^). The *KRAS* G12C mutation favors the active form of *KRAS*, which results in an abnormally high amount of GTP-bound *KRAS*, leading to an overactivation of downstream oncogenic pathways and uncontrolled cell growth. It was only in 2013 that Ostrem *et al*^28^ were able to identify an allosteric site that allows for the binding of the *KRAS* G12C inhibitors and the advancement in their development.

In 2021, sotorasib was the first *KRAS* G12C inhibitor approved by the US Food and Drug Administration (FDA) after one prior line of therapy.^41^ By covalently binding to the mutant cysteine residue of the 12th codon, sotorasib is able to lock *KRAS* in an inactive GDP-bound state and inhibit the signaling and growth of cancer.^47^^,^^48^ Due to the financial cost of sotorasib and the developed resistance, adagrasib was subsequently approved for market use. Its phase 2 results showed patients with *KRAS* G12C-mutated NSCLC were able to have an objective response rate (ORR) of 42.9 %, a median duration of response (DOR) of 8.5 months, and a median PFS of 6.5 months (Table 3).^47^^,^^49^^,^^50^^,^^51^ The current guideline now recommends that clinicians offer single-agent sotorasib or adagrasib to patients with advanced NSCLC who have a *KRAS* G12C mutation and have received prior systemic therapy, including chemotherapy and anti-PD-L1 therapy (Table 2).^52^

**Table 3:** Efficacy of approved treatments for *KRAS-*mutated NSCLC.

Therapy	ORR (%)	Ref.
Immunotherapy alone	37	50
Chemotherapy alone	35	50
Chemo-immunotherapy	40–46	50,51
Sotorasib	37.1	47
Adagrasib	42.9	49

*KRAS*: Kirsten rat sarcoma viral oncogene homolog; NSCLC: Non-small cell lung carcinoma; ORR: Objective response rate; Ref: Reference.

## Resistance to *KRAS* G12C inhibitors

**Fig. 3:**
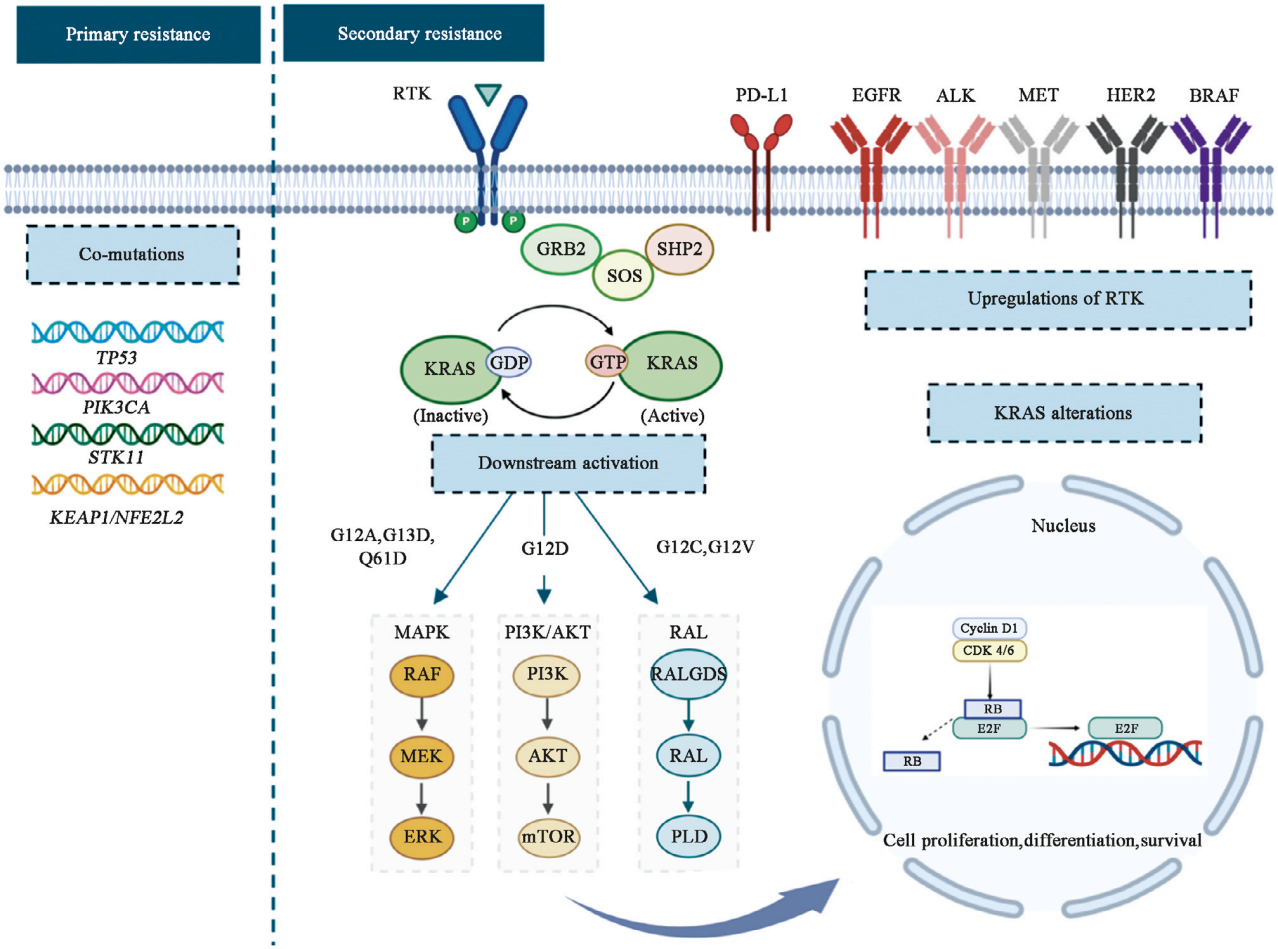
*KRAS* signaling pathway with potential mechanisms of resistance. Primary resistance is most common due to co-occurring mutations with *TP53, PIK3CA, STK11*, and *KEAP1/NFE2L2*. Secondary resistance can arise through additional driver mutations that lead to RTK upregulation, *KRAS* alterations, and downstream pathway activations. AKT: Protein kinase B; *ALK*: Anaplastic lymphoma kinase; BRAF: V-raf murine sarcoma viral oncogene homolog B1; CDK: Cyclin-dependent kinase; E2F: Early region 2 binding factor; *EGFR*: Epidermal growth factor receptor; ERK: Extracellular signal-regulated kinase; G12A: Glycine-to-alanine substitution at codon 12; G12C: Glycine-to-cysteine substitution at codon 12; G12D: Glycine-to-aspartic acid substitution at codon 12; G12V: Glycine-to-valine substitution at codon 12; G13D: Glycine-to-aspartic acid substitution at codon 13; Q61D: Glutamine-to-aspartic acid substitution at codon 61; GDP: Guanosine diphosphate; GRB2: Growth factor receptor-bound protein 2; GTP: Guanosine triphosphate; HER2: Human epidermal growth factor receptor 2; *KEAP1*: Kelch-like ECH-associated protein 1; *KRAS*: Kirsten rat sarcoma viral oncogene homolog; MAPK: Mitogen-activated protein kinase; MEK: Mitogen-activated protein kinase (MAPK) kinase; MET: Mesenchymal-epithelial transition; mTOR: Mammalian target of rapamycin; NFE2L2: Nuclear factor erythroid 2-related factor 2; P: Phosphorylation; PD-L1: Programmed death-ligand 1; PI3K: Phosphoinositide-3-kinase; *PIK3CA*: Phosphatidylinositol-4,5-bisphosphate 3-kinase catalytic subunit alpha; PLD: Phospholipase D; RAF: Rapidly accelerated fibrosarcoma; RAL: Ras-like; RALGDS: Ral guanine nucleotide dissociation stimulator; RB: Retinoblastoma tumor suppressor protein; RTK: Receptor tyrosine kinase; SHP2: Src homology region 2 domain-containing phosphatase 2; SOS: Son of sevenless; STK11: Serine/threonine kinase 11; *TP53*: Tumor protein 53.

Given the inherent complexity of lung cancer, resistance to the current therapies available is virtually inevitable. The resistance to *KRAS*^G12C^ inhibitors can be largely categorized into primary/innate resistance and secondary/acquired resistance (Fig. 3).

### Primary resistance

Although *KRAS* mutations in NSCLC have been considered mutually exclusive driver mutations, there has been evidence of co-occurring mutations in correlation with different *KRAS* mutation subtypes.^18^^,^^53^ The most frequent co-occurring mutations were found in *TP53* (42 %), *STK11* (29%), and *KEAP1*/nuclear factor erythroid 2-related factor 2 (*NFE2L2*) (27 %).^54^ The presence of these innate co-mutations has been shown to affect efficacy. *STK11*/liver kinase B1 (*LKB1*) alterations have been identified as a major driver of primary resistance to PD-1 blockade and are associated with inferior treatment outcomes.^27^ Furthermore, *KEAP1*/*NFE2L2* co-mutation can cause a shorter duration of response to chemotherapy and immunotherapy, leading to shorter survival.^53^^,^^54^ Co-alterations in *STK11/LKB1* and *KEAP1/NFE2L2* have been associated with a suppressed anti-tumor immune microenvironment, which may contribute to primary resistance not only to immunotherapy but also potentially limit the effectiveness of KRAS G12C inhibitors, though direct evidence is still emerging. KRAS G12C inhibitors were designed to covalently bind the cysteine residue unique to the G12C variant, locking KRAS in an inactive state and preventing RAF activation. This directly suppresses MAPK/ERK signaling, which is the dominant oncogenic signaling cascade in most *KRAS*-driven tumors. However, the G12C variant has also been shown to engage in the RALA/B pathway, which is not dependent on RAF activity.^17^ As a result, resistance may occur due to the residual oncogenic signaling through the RALA/B pathway.

### Secondary resistance

Despite the groundbreaking discovery and the clinical benefits observed with *KRAS* G12C inhibitors, acquired resistance occurred in most patients. Reita *et al*^55^ have classified these secondary resistance mechanisms into two groups: "on-target" and "off-target". The "on-target" group is associated with mutations in the binding region of the *KRAS* G12C, and the "off-target" mechanisms include genetic events that can occur in the upstream regulation of *KRAS* or downstream and parallel signaling pathways. The current generation of *KRAS* G12C inhibitors are selective inhibitors of the G12C mutant and, therefore, do not inhibit wild-type RAS. However, wild-type and mutant *KRAS* could coexist in the same cell and work together in a feedback mechanism to reactivate RAS signaling when one of the two *KRAS* pathways is blocked. In addition, *KRAS* G12C inhibition can be overcome through feedback activation of the upstream or downstream regulators within the RTK-KRAS-MAPK cascade,^55^ resulting in off-target resistance. Furthermore, the emergence of secondary resistance can be driven by concurrent genomic alterations in bypass pathways, as exemplified by mutations in EGFR or ALK.

## Therapeutic options for *KRAS*-mutated NSCLC

**Fig. 4:**
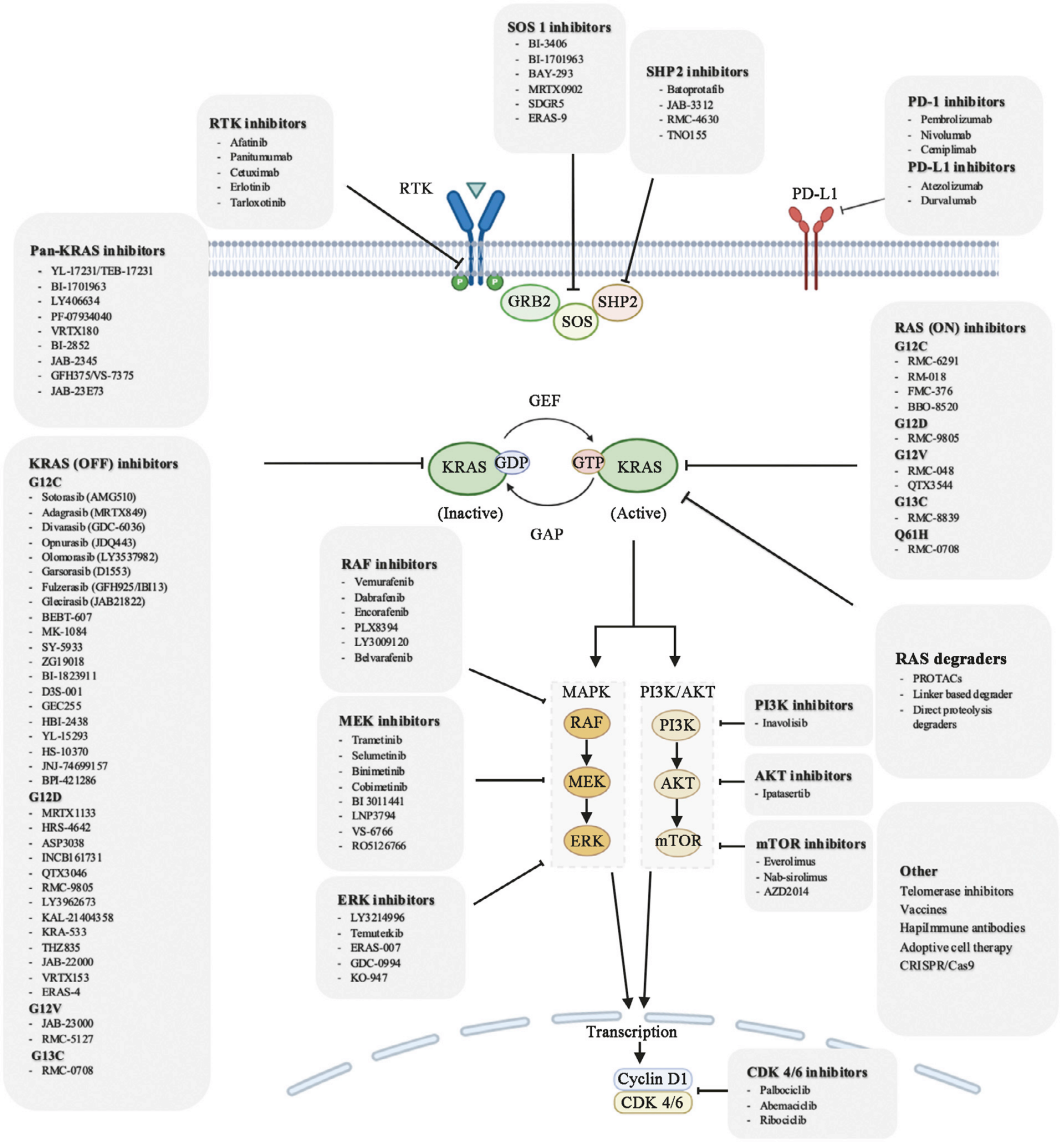
Overview of *KRAS* pathway and drugs to inhibit *KRAS*-mutated tumors. *KRAS* activity can be targeted either directly using *KRAS* inhibitors or indirectly by inhibiting its upstream activators or downstream signaling pathways. Other possible ways to inhibit *KRAS* indirectly include vaccination, PROTACs, siRNA, and adoptive cell therapies. AKT: Protein kinase B; CDK 4/6: Cyclin-dependent kinase 4/6; CRISPR/Cas9: Clustered regularly interspaced short palindromic repeats/CRISPR-associated protein 9; ERK: Extracellular-regulated protein kinase; G12C: Glycine-to-cysteine substitution at codon 12; G12D: Glycine-to-aspartic acid substitution at codon 12; G12V: Glycine-to-valine substitution at codon 12; G13C: Glycine-to-cysteine substitution at codon 13; GEF: Guanine nucleotide exchange factor; GAP: GTPase-activating protein; GDP: Guanosine diphosphate; GTP: Guanosine triphosphate; GRB2: Growth factor receptor-bound protein 2; JAB: Janus kinase-binding protein; *KRAS*: Kirsten rat sarcoma viral oncogene homolog; MAPK: Mitogen-activated protein kinase; MEK: Mitogen-activated protein kinase kinase; mTOR: Mammalian target of rapamycin; PD-1: Programmed death-1; PD-L1: Programmed death ligand 1; PI3K: Phosphoinositide 3-kinase; Q61H: Glycine- to-histidine substitution at codon 61; RAF: Raf kinase; RAS: Rat sarcoma; RTK: Receptor tyrosine kinase; SHP2: Src homology 2 domain-containing protein tyrosine phosphatase 2; SOS: Son of sevenless; PROTACs: Proteolysis-targeting chimeras.

Various strategies to target *KRAS* mutation can be approached through direct inhibition, which focuses on specifically blocking the mutant protein, or indirect inhibition, which aims to disrupt the downstream signaling pathways influenced by *KRAS* activation (Fig. 4).

### Direct inhibition of KRAS

Direct inhibitors include the current FDA-approved *KRAS* G12C inhibitors, sotorasib, and adagrasib, which covalently bind to and trap the mutant *KRAS* in its inactive, GDP-bound state, known as RAS (OFF) inhibitors. In addition, many other *KRAS* (OFF) inhibitors are under development, as well as emerging RAS (ON) inhibitors that target the active GTP-bound state of *KRAS*. These RAS (ON) inhibitors recruit cyclophilin A and form a tri-complex. Cyclophilin A cannot interact with *KRAS* by itself, but in a complex formation with the inhibitors, it can occlude the effector binding sites of *KRAS* G12C.^56^ Most G12, G13, and Q61 mutants are reported to be in the GTP state and prevent GTP hydrolysis. As a result, RAS (ON) inhibitors are expected to be more effective than RAS (OFF) inhibitors. They may even overcome acquired resistance developed after treatment with RAS (OFF) inhibitors. RMC-6235 is the first RAS multi (ON) inhibitor that can treat cancers of *KRAS* mutations, especially in *KRAS* G12 mutant genotypes.^45^ It can inhibit both mutant and wild-type RAS-GTP. RM-018 is another *KRAS* G12C (ON) inhibitor that not only targets G12C but remains effective in the presence of Y96D, one of the resistance mechanisms that can develop following treatment with sotorasib or adagrasib.^57^ Table 4 summarizes the clinical development of KRAS inhibitors for lung cancer.^45^^,^^48^^,^^49^^,^^58^, ^59^, ^60^, ^61^, ^62^, ^63^, ^64^, ^65^, ^66^, ^67^, ^68^, ^69^, ^70^, ^71^, ^72^, ^73^, ^74^, ^75^, ^76^, ^77^, ^78^, ^79^, ^80^, ^81^, ^82^, ^83^, ^84^, ^85^, ^86^, ^87^, ^88^, ^89^, ^90^, ^91^, ^92^, ^93^, ^94^, ^95^, ^96^, ^97^, ^98^, ^99^, ^100^, ^101^, ^102^, ^103^, ^104^, ^105^, ^106^, ^107^, ^108^, ^109^

**Table 4:** Current status of *KRAS* inhibitors for lung cancer.

Type of *KRAS* inhibitor	Drug target	Drug	Source	Trial	Status	Results	TRAEs (%)	Reference
*KRAS*- **(OFF) inhibitors**	G12C	Sotorasib (AMG510)	Amgens; USA	NCT04303780	Approved in the USA, Europe, and Japan	ORR 28.1 %; DCR 82.5 %; mDOR 8.6 mo; mOS 10.6 mo; mPFS 5.6 mo;	Any grade: 98 % Grade 3+: 33 % (diarrhea 12 %, ↑ALT 8 %, ↑AST 5 %); Grade 5: <1 % (interstitial lung disease)	^48^
		Adagrasib (MRTX84)	Bristol Myers Squibb; USA	NCT03785249	Approved in the USA	Phase 2: ORR 42.9 %, mPFS 6.5 mo, mDOR 8.5 mo, mOS 12.6 mo; Phase 3: ORR 31.9 %, mDOR 8.31 mo, mPFS 5.49 mo	Phase 2 Grade 1–2: 52.6 % Grade3+: 44.8 %; Phase 3 Any grade: 94 % Grade 3+: 47.0 %	^49^^,^^58^
		Divarasib (GDC-6036)	Hoffmann-La Roche; USA	NCT06497556	Phase 3; recruiting	ORR 53.4 %, mPFS 13.1 mo	Any grade: 93 %; Grade 3: 11 %; Grade 4: 1 %; no DLT	^59^
		Opnurasib (JDQ443)	Novartis; USA	NCT05132075	Phase 3; discontinued	ORR 57.1 %	Any grade: 75 %; Grade 3: 5.9 %; Grade 4–5: none	^60^^,^^61^
		Olomorasib (LY3537982)	Eli Lilly; USA	NCT04956640	Phase 1/2; recruiting	G12Ci-naïve: ORR 40 %; mPFS 9 mo G12Ci-pretreated: ORR 39 %, DCR 73 %, mPFS 6 mo	Any grade: 62 % (diarrhea 24 %, fatigue 10 %, nausea 10 %); Grade 3+: 5 %	^62^
		Garsorasib	InventisBio; China	NCT05383898	Phase 2; completed	ORR 50 % (61/153) mPFS 8.2 mo	Any grade: 95 %; Grade 3+: 50 % (↑AST 17 %, ↑ALT 15 %, ↑γ-GGT 23 %)	^63^
		Fulzerasib (GFH925/IB351)	Innovent Biologics; China	NCT05005234	Phase 2; recruiting; approved in China	ORR 49.1 % mPFS 9.7 mo	Anemia 44.8 %, ↑ALT 28.4 %, ↑AST 27.6 %, asthenia 26.7 %, proteinuria 25 %	^64^
		BEBT-607	BeBetterMed, Inc; China	NCT06117371	Phase 1; recruiting	No data	No data	-
		MK-1084	Merck; USA	NCT05067283	Phase 1; recruiting	ORR 38 %	Any grade: 58 %	^65^
		SY-5933	Shouyao Holdings, China	NCT06006793	Phase 1; recruiting	No data	No data	^65^
		Glecirasib (JAB21822)	Jacobio; China	NCT05009329	Phase1/2; active	ORR 47.9 %; DCR 86.3 %; mPFS 8.2 mo; mOS 13.6 mo; mDOR not been reached	Any grade: 97.5 %; anemia 56.3 %, ↑bilirubin 48.7 %, ↑ALT/AST 35.3 % Grade 3–4: 39.5 %; Grade 5: none	^66^
		ZG19018	SuZhou Zelgen; China	CTR20220296, NCT06237400	Phase 1/2; recruiting	No data	Any grade: 92.9 %; nausea 42.9 %, diarrhea 42.9 %, vomiting 35.7 %, ↑ALT 35.7 %, ↑γ-GGT 35.7 %, ↑AST 35.3 %	^67^
		BI-1823911	Boehringer Ingelheim; Germany	NCT04973163	Phase 1a/b; active	No data	No data	^68^
		D3S-001	D3 Bio; China	NCT5410145	Phase 1; recruiting	G12Ci-naïve: ORR 73.5 % (25/34) G12Ci-pretreated: ORR 30.0 % (6/20), DCR 80.0 % (16/20)	Grade 3: 16.7 % in G12Ci-naïve,10 % in G12Ci-pretreated; Grade 4 or 5: none; no DLT; MTD not reached	^69^
		GEC255	GenEros Biopharma; China	NCT05768321, CTR20212486	Phase 1; recruiting	Overall ORR 76.9 % (10/13), DCR 92.3 % (12/13); 600 mg dose group: ORR 83.3 % (5/6), DCR 100 % (6/6)	Grade 1 or 2: 93 % (diarrhea 56.3 %, ↑ALT 37.5 %, rashes 25 %, anemia 25 %); no DLT; MTD not reached	^70^

								
		HBI-2438	Huyabio; China	NCT05485974	Phase 1; recruiting	No data	No data	^71^
		YL-15293	Shanghai Yingli; China	NCT05119933	Phase 2; unknown	No data	No data	^72^
		HS-10370	Jiangsu Hansoh; China	NCT05367778	Phase 1/2; recruiting	Overall (*n* = 48) ORR 54.2 %, DCR 93.8 %, mDOR 13.5 mo, mPFS 13.5 mo; first-line (≥400 mg QD, *n* = 22): ORR 72.7 %, DCR 95.5 %, mDOR 9.7 mo, mPFS 12.5 mo; second-line (≥400 mg QD, *n* = 19): ORR 57.9 %, DCR 94.7 %, mDOR 20.3 mo, mPFS 12.5 mo	Any grade: 81 % (↑AST 28.6 %, ↑ALT 28.6 %, anemia 25.4 %, diarrhea 17.5 %, weight gain 14.3 %, decreased appetite 12.7 %; Grade 3: 22.2 % (↑AST, QT prolong, ↑blood creatine kinase); Grade 4–5: none	^73^^,^^74^
		JNJ-74699157	Janssen; USA	NCT04006301	Phase 1; completed	No significant clinical benefit observed	Enrollment stopped at 10 patients due to dose-limiting grade 3–4: ↑blood creatinine phosphokinase	^75^
		BPI-421286	Betta Pharmaceuticals; China	NCT05315180	Phase 1; unknown	No data	No data	^76^
	G12D	MRTX1133	Mirati; USA	NCT05737706	Phase 1/2; terminated	Study was terminated prior to phase 2 initiating	No Data	^77^
		HRS-4642	Jiangsu Hengrui; China	NCT05533463	Phase 1; recruiting	One patient at 200 mg had partial response	Grade 3+: 33.3 % (hypercholesterolemia 16.7 %, ↑lipase 11.1 %, anemia 11.1 %); no DLT; MTD not reached	^78^^,^^79^
		ASP3082	Astellas Pharma; USA	NCT05382559	Phase 1; recruiting	No data	Any grade: 69.4 % (fatigue 15.3 %, infusion-related reactions 14.3 %, pruritus 9.2 %)	^80^
		INCB161731	Incyte; USA	NCT06179160	Phase 1; recruiting	No data	No data	^81^
		QTX3046	Quanta Therapeutics; USA	NCT06428500	Phase 1/1b; recruiting	No data	No data	^82^
		LY3962673	Eli Lilly; USA	NCT06586515	Phase 1/1b; recruiting	No data	No data	^83^
		KAL-21404358	Tsinghua University; China	N/A	Pre-clinical	No data	No data	^84^
		KRA-533	National Cancer Institute; USA	N/A	Pre-clinical	No data	No data	^85^
		THZ835	Tsinghua University; China	N/A	Pre-clinical	No data	No data	^72^
		JAB-22000	Jacobio; China	N/A	Pre-clinical	No data	No data	^86^
		VRTX153	VRise Therapeutics; USA	N/A	Pre-clinical	No data	No data	^65^^,^^87^
		ERAS-4	Erasca; USA	N/A	Pre-clinical	No data	No data	^74^
	G12V	JAB-23000	Jacobio; China	N/A	Pre-clinical	No data	No data	^88^
		RMC-5127	Revolution Medicines; USA	N/A	IND-enabling	No data	No data	^89^^,^^90^
	Q61H	RMC-0708	Revolution Medicines; USA	N/A	IND-enabling	No data	No data	^72^
	G13C	RMC-8839	Revolution Medicines; USA	N/A	IND-enabling	No data	No data	^91^
**RAS- (ON) inhibitors**	G12C	RMC-6291	Revolution Medicines; USA	NCT05462717	Phase1/1b; active	G12Ci-naïve: ORR 43 %; DCR 100 % G12Ci-pretreated: ORR 50 %; DCR 100 %	Any grade: Diarrhea 29 %, nausea 27 %, QT prolonged 25 % Grade 4–5: none	^92^
		RM-018	Revolution Medicines; USA	N/A	Pre-clinical	No data	no data	^93^
		FMC-376	Frontier Medicines; USA	NCT06244771	Phase 1/2; recruiting	No data	No data	^94^
		BBO-8520*	BridgeBio; USA	NCT06343402	Phase 1a/1b; recruiting	No data	No data	^95^^,^^96^

								
	G12D	Zoldonrasib (RMC-9805)	Revolution Medicines; USA	NCT06040541	Phase 1/1b; recruiting	ORR 61 %; DCR 89 %	Grade 1–3: 74 % (G1: 54 %, G2: 18 %, G3: 2 %); nausea 39 %, diarrhea 24 %, vomiting 17 %, rash 12 % Grade 4–5: none	^97^
	G12V	RMC-048	Revolution Medicines; USA	N/A	Pre-clinical	No data	No data	^72^
	G13C	RMC-8839	Revolution Medicines; USA	N/A	Pre-clinical	No data	No data	^72^
	Q61H	RMC-0708	Revolution Medicines; USA	N/A	Pre-clinical	No data	No data	^72^
**Multi/Pan-RAS inhibitors**	*KRAS* G12C & SOS1	BI-1701963	Boehringer Ingelheim; Germany	NCT04111458	Phase 1; active	No data	Any grade: 18 % (diarrhea, fatigue, decreased platelet count 14 %); Grade 3+: 10.7 % (G3 hypertension, G3 duodenal obstruction, G4 ↓platelet count)	^98^
	Pan-*KRAS*	LY4066434	Eli Lilly; USA	NCT06607185	Phase 1; recruiting	No data	No data	^99^
		VRTX180	VRise Therapeutics; USA	N/A	Pre-clinical	No data	No data	^65^
		PF-07934040	Pfzier; USA	NCT06447662	Phase 1; recruiting	No data	No data	-
		JAB-23E73	Jacobio; China	NCT06973564	Phase 1/2; not yet recruiting	No data	No data	^100^
		JAB-23425	Jacobio; China	N/A	Pre-clinical	No data	No data	^101^
	*KRAS* G12C, G12D, G12V, G13D	BI-2865	Boehringer Ingelheim; Germany	N/A	Pre-clinical	No data	No data	-
	*KRAS* G12V- preferring	QTX3544	Quanta Therapeutics; USA	NCT06715124	Phase 1; recruiting	No data	No data	^102^
	*KRAS* G12D- preferring	GFH375/VS-7375	GenFleet; China	NCT06500676	Phase 1/2 in China; recruiting	ORR 42 %; DCR 83 %	Mainly Grade 1/2: (vomiting, nausea, anemia); Grade3+: decreased neutrophil count 8 %, diarrhea 5 %; no DLT	^103^^,^^104^
		QTX3034	Quanta Therapeutics; USA	NCT06227377	Phase 1; recruiting	No data	No data	^105^
		BI-2852	Boehringer Ingelheim; Germany	N/A	IND-enabling	No data	No data	^106^
	*KRAS*, HRAS,NRAS	Daraxonrasib (RMC-6236)	Revolution Medicines; USA	NCT05379985	Phase 1/1b; recruiting	ORR 52 % (15/29); DCR 83 % (24/29)	Any grade: rash 52 %, diarrhea 21 %, nausea 21 %, vomiting 15 %	^45^^,^^107^
		YL-17231/TEB-17231	Shanghai YingLi; China	NCT06096974 NCT06078800	Phase 1; recruiting	No data	No data	^108^
		RMC-7977	Revolution Medicines; USA	N/A	Pre-clinical	*In vivo*: 83 % mean tumor regression following 28 days of treatment	No data	^109^

ALT: Alanine aminotransferase; AST: Aspartate aminotransferase; DCR: Disease control rate; DLT: Dose-limiting toxicities; G12C: Glycine-to-cysteine substitution at codon 12; G12D: Glycine-to-aspartic acid substitution at codon 12; G12V: Glycine-to-valine substitution at codon 12; G13C: Glycine-to-cysteine substitution at codon 13; G13D: Glycine-to-aspartic acid substitution at codon 13; G12Ci: G12C inhibitor; *HRAS*: Harvey rat sarcoma viral oncogene homolog; IND-enabling: Investigational new drug-enabling; *KRAS*: Kirsten rat sarcoma viral oncogene homolog; mDOR: Median duration of response; mo: Months; mOS: Median overall survival; mPFS: Median progression-free survival; MTD: Maximum tolerated dose; N/A: Not available; *NRAS*: Neuroblastoma rat sarcoma viral oncogene homolog; ORR: Objective response rate; Q61H: Glutamine-to-histidine substitution at codon 61; QD: Every day; SOS1: Son of sevenless 1; TRAEs: Treatment related adverse events; γ-GGT: Gamma-glutamyl transferase. *Targets both *KRAS* G12C ON and OFF state.

Inhibitors specific to the other major point mutations, G12D, G12V, and Q61H, are also in clinical trials (Table 4). Furthermore, pan-*KRAS* inhibitors that do not discriminate between *KRAS* mutants are also being developed to cover a broader range of *KRAS* G12C, G12D, and G12V mutations (Table 4). BI-2865 is the first pan-*KRAS*-selective inhibitor that only selectively binds to *KRAS*, not other RAS family proteins, and can target most *KRAS* mutants.^44^ GFH375/VS-7375 is a dual *KRAS* G12D inhibitor that works on both ON and OFF states and showed strong potency in intracranial tumor models. It is undergoing a phase 1/2 study in China to evaluate its efficacy against NSCLC and other solid tumors.

#### Current status of KRAS G12C inhibitors

In addition to the FDA-approved sotorasib and adagrasib, most *KRAS* inhibitors in development are in the early stages of clinical trials and lack sufficient clinical trial data. Therefore, we focus our review on the selected next-generation inhibitors at the later clinical trial stages with published results.

##### Divarasib (GDC-6036)

Divarasib has been shown to be 5 to 20 times more potent and 50 times more selective than sotorasib and adagrasib.^59^ In its phase 3 trial, there were fewer dose reductions and discontinuation of treatments, with gastrointestinal events being the primary adverse effect. The ORR observed in NSCLC patients was 56.4 %, and the median PFS was 13.7 months,^59^ which seems slightly better than sotorasib and adagrasib.

##### Opnurasib (JDQ443)

Like other *KRAS* G12C inhibitors, opnurasib (JDQ443) traps *KRAS* G12C in the inactive, GDP-bound state. When used as neoadjuvant therapy in a phase II study, opnurasib showed an ORR of 57.1 % at the recommended phase 2 dose.^61^ Another phase 1b/II trial showed that when used as a monotherapy, opnurasib demonstrated an ORR of 41.7 % across all dose levels and 54.5 % at the regular dose of 200 mg twice a day.^62^ While opnurasib showed promising results, Novartis has decided to discontinue its phase 3 trial and its development due to increasing options available in the market.^60^

##### Fulzerasib (IBI315/GFH925)

Across the globe, an irreversible *KRAS* G12C inhibitor developed in China by Innovent Biologics is IBI315, also known as GFH925/fulzerasib. In its phase 2 trial, fulzerasib showed an ORR of 49.1 %, a disease control rate of 90.5 %, and a median PFS of 9.7 months.^110^ The results led to its approval by China's National Medical Products Administration (NMPA) in August 2024 as the first *KRAS* G12C inhibitor in China for patients with *KRAS* G12C-driven NSCLC who have received at least one prior systemic therapy.^64^

Other *KRAS* inhibitors against the different RAS variants in their early clinical trial stages are also listed in Table 4, along with their associated safety and efficacy data.

#### RAS degraders

Another way to directly inhibit *KRAS* is to degrade it before it initiates the downstream signaling pathways. Degradation is considered to be more potent than direct inhibition as it allows for a more sustained effect on downstream signaling. Furthermore, it can kill cancer cells while sparing those without the genetic *KRAS* mutations. RAS degraders include proteolysis-targeted chimeras (PROTACs), linker-based degraders, and direct proteolysis degraders.

PROTACs work by bringing the ubiquitin ligase to the target protein and marking it for degradation in a proteasome-dependent manner. Early PROTACs use covalent *KRAS* binders that result in irreversible inhibition. LC-2 is the first PROTAC capable of inducing *KRAS* G12C degradation by recruiting von Hippel-Lindau (VHL) E3 ligase complex.^40^ Recent development focuses on noncovalent *KRAS* binders that can engage and degrade a wider range of *KRAS* variants beyond G12C. Among these, ASP3082 is a potent and selective KRAS G12D degrader that has demonstrated growth inhibition activity in *KRAS* G12D-mutant pancreatic cancer cells while sparing *KRAS* wild-type cancer cells. Another notable example is ACBI3, a pan-KRAS degrader capable of targeting and degrading the majority of oncogenic KRAS variants.^111^^,^^112^

Direct proteolysis degraders include RAS/Rap1-specific endopeptidases (RRSP) produced by the bacterium *Vibrio vulnificus*.^16^ Another tumor-targeting *KRAS* degrader (TKD) is a KRAS nanobody that can induce pan-*KRAS* degradation in cancer cells via a lysosome-dependent degradation pathway. This offers a novel mechanism of action that bypasses the need for proteasomal degradation pathways commonly exploited by other targeted therapies.^46^

### Indirect Inhibition of *KRAS*

As mentioned previously, resistance to treatment often occurs due to the feedback activation of either the upstream or downstream regulators within the RTK-KRAS-MAPK cascade and parallel pathways (Fig. 4). Indirect inhibition targets the critical molecules in these regulating pathways.

#### Upstream inhibition: RTK, SOS1, and SHP2 inhibitors

As an important converter of the GDP to GTP bound state, GEF consists of a complex of proteins, including SOS1, SHP2, and GRB2, is activated by upstream signaling from RTKs. This activation is, in turn, inhibited by RTK inhibitors. SOS1 has two binding sites for *KRAS*, an allosteric binding site, and a catalytic binding site. It works by activating the GDP-bound RAS at its catalytic binding site to promote the exchange of GDP for GTP as well as bringing GTP-bound RAS to its allosteric site that potentiates its own GEF function, promoting a positive feedback loop.^113^ By inhibiting SOS1 and SHP2, the GDP-GTP exchange is blocked, keeping KRAS in a constitutively inactive state. As seen in Table 5, there are a handful of clinical trials testing the efficacy of *KRAS* G12C inhibitors with SHP2 inhibitors and SOS1 inhibitors.

**Table 5:** Combination therapy with *KRAS* inhibitors for lung cancer.

Target	Inhibitor	*KRAS* inhibitor	Trial	Phase and status
RTK	Cetuximab	Opnurasib	NCT05358249	1/2; Active
		Glecirasib	NCT05002270	1/2; Recruiting
		Divarasib	NCT04449874	1; Recruiting
		Olomorasib	NCT04956640	1; Recruiting
	Erlotinib	Divarasib	NCT04449874	1; Recruiting
	Afatinib	Sotorasib	NCT04185883	1; Recruiting
	Panitumumab	Sotorasib	NCT05638295	2; Recruiting
	Osimertinib	Sotorasib	NCT04959981	1; Completed
SHP2	ERAS-601	Sotorasib	NCT04959981	1; Completed
	RMC-4630	Sotorasib	NCT04185883	1; Recruiting
	TNO155	Sotorasib	NCT04185883	1; Recruiting
		Adagrasib	NCT04330664	1/2; Active
		Opnurasib	NCT04699188	1/2; Active
	GDC-1971	Divarasib	NCT04449874	1; Recruiting
	JAB-3312	Glecirasib	NCT05288205	1/2; Recruiting
	RMC-4630	Sotorasib	NCT05054725	2; Unknown status
SOS1	BI 1701963	Sotorasib	NCT04185883	1; Recruiting
		BI 1823911	NCT04973163	1; Active
	MRTX0902	Adagrasib	NCT05578092	1/2; Recruiting
Wild-type RAS	RMC-6236	RMC-6291	NCT06128551	1; Recruiting
MEK	Avutometinib	Adagrasib	NCT05375994	1/2; Active
	Trametinib	Opnurasib	NCT05358249	1/2; Active
		Sotorasib	NCT04185883	1; Recruiting
ERK	ERAS-007	Sotorasib	NCT04959981	1; Completed
PI3K	Inavolisib	Divarasib	NCT04449874	1; Recruiting
mTOR	Everolimus	Sotorasib	NCT04185883	1; Recruiting
	Nab-sirolimus	Adagrasib	NCT05840510	1/2; Active
CDK4/6	Palbociclib	Adagrasib	NCT05178888	1; Active
		Sotorasib	NCT04185883	1; Recruiting
	Ribociclib	Opnurasib	NCT05358249	1/2; Active
ULK1/2	DCC-3116	Sotorasib	NCT04892017	1/2; Recruiting
PARP	Olaparib	Adagrasib	NCT06130254	1; Recruiting
CXCR1/2	Ladarixin	Adagrasib	NCT05815173	1; Recruiting
VEGF	Bevacizumab	Divarasib	NCT04449874	1; Recruiting

This table includes indirect *KRAS* inhibitors being tested with *KRAS* inhibitors in clinical trials. Immune checkpoint inhibitors are not included. CDK: Cyclin-dependent kinase; CXCR: C-X-C chemokine receptor; ERK: Extracellular signal-regulated kinase; *KRAS*: Kirsten rat sarcoma viral oncogene homolog; MEK: Mitogen-activated protein kinase; mTOR: Mammalian target of rapamycin; PARP: Poly (ADP-ribose) polymerase; PI3K: Phosphoinositide-3-kinase; RAS: Rat sarcoma; RTK: Receptor tyrosine kinase; SHP2: Src homology region 2 domain-containing phosphatase 2; SOS1: Son of sevenless 1; ULK: Unc-51 like autophagy activating kinase; VEGF: Vascular endothelial growth factor.

#### Downstream inhibition: RAF/MEK/ERK inhibitors

Given the heterogeneity of oncogenic RAS mutations, targeting downstream signaling pathways offers a strategy to circumvent this heterogeneity. The two major direct downstream signaling pathways of RAS are the MAPK and PI3K signaling pathways.^114^ Once RAF proteins are activated, they phosphorylate MEK, which, in turn, activates extracellular signal-regulated kinases 1 and 2 (ERK1/2). Activated ERK1/2 will then translocate into the nucleus, where they regulate transcription factors involved in cell proliferation, differentiation, survival, and migration. Inhibition of the RAF/MEK/ERK pathway can attenuate these downstream signals, bypassing the constitutive upstream KRAS activation.

#### Parallel pathway inhibition: PI3K/AKT/mTOR inhibitors

Activation of the PI3K/AKT signaling pathway has been identified as a key mechanism contributing to resistance not only to chemotherapy and other targeted therapies but also to *KRAS* G12C inhibitors.^115^ While it is well known that the PI3K/AKT pathway is frequently mutated in human cancers, the clinical efficacy with developed single-agent PI3K inhibitors has been modest. The inhibition has been unpredictable, likely due to its complexity, which includes many feedback loops and interactions with other RAS-regulated pathways. In cancer cell lines resistant to sotorasib, Chan et al^115^ found that p21-activated kinases (PAK) and PI3K pathways were activated, and together, they formed a mutual positive regulatory loop that mediates sotorasib resistance. When used together, an FDA-approved PI3K inhibitor, alpelisib, was reported to work synergistically with sotorasib in treating other cancers with *KRAS* G12C mutation.

### Telomerase inhibitors

While *KRAS* mutation results in continuous activation of the RAS/MEK/ERK pathways, it is reported that this activation also upregulates telomerase. The RAS pathway activation upregulates telomerase activity by activating telomerase reverse transcriptase (TERT) transcription.^116^ Telomerase is an enzyme that synthesizes and maintains telomeres, repeatable DNA protein complexes that serve as protective caps at the ends of chromosomes, essential for chromosome stability. Telomerase is normally inactive in somatic cells, allowing telomere length to decrease with every cell division as part of the natural aging cycle. Cancer cells are known to have evolved to overcome senescence through mechanisms such as activating telomerase to maintain telomere lengths, so cancer cells can divide indefinitely.^117^ Additionally, elevated expression of TERT was observed in *KRAS*-mutated NSCLC compared to wild-type *KRAS*.^35^

Since its first discovery, telomerase inhibitor BIBR1532 has shown anti-cancer properties.^118^ A study has demonstrated that BIBR1532 not only shortens telomere length and inhibits mutant *KRAS*-induced proliferation but also sensitizes *KRAS*-expressing lung adenocarcinoma (Calu-3) cells to chemotherapeutic drugs.^35^ Another study showed that BIBR1532 enhances the radiosensitivity of NSCLC in a synergistic way through increasing telomere dysfunction and impairing DNA damage repair by inhibiting the ataxia telangiectasia mutated (ATM)/checkpoint kinase 1 (CHK1) pathway.^119^ Targeting telomerase could be a promising therapeutic approach for patients with *KRAS* mutant NSCLC.

### New immunological approaches

Given that *KRAS* mutations drive immune evasion, treatments leveraging immunologic approaches are in development. These include adoptive cell therapies, cancer vaccines, and HapImunne antibodies.

#### Adoptive cell therapies

While cancer mutations may occur due to an incidental event, their accumulation is largely driven by the ability of these cancer cells to evade immune surveillance. Therefore, reconstructing and re-educating the immune system has been a hot research topic to harness the body's immune defenses to target and eliminate tumor cells effectively. First-generation adoptive cell therapies (ACT) use autologous peripheral lymphocytes that lack specific targeting and killing abilities. This approach led to the emergence of T cell receptor T cell therapy (TCR-T), chimeric antigen receptor-T cells (CAR-T), chimeric antigen receptor-macrophages (CAR-M), and chimeric antigen receptor-natural killer cells (CAR-NK). Unlike antibodies that recognize and need to bind to intact cell surface proteins, T cells can determine the mutated proteins via TCR recognition of neoantigen peptides presented by the human leukocyte antigen (HLA) class I on these cancer cells. An engineered TCR has been able to recognize peptides from *KRAS* G12D mutant.^120^ CAR is an artificial combined TCR consisting of immunoglobulin, co-stimulation factors, and CD3. They can be activated without CD3 and, therefore, can be made into macrophages and natural killer cells. Unlike TCR, which can only recognize HLA-presented peptides, CAR can recognize antigens on the cell surface, targeting glycoproteins and glycolipids as extended antigens.

#### Cancer vaccines

Vaccines are another avenue of exploration in the field to combat *KRAS* mutation in lung cancer. The cancer vaccines can induce cancer-specific long-term memory T cells and promote *KRAS* anti-tumor immunity by providing oncogenic *KRAS* neoantigens to MHC molecules. Several types of vaccines in development include peptide-, messenger RNA (mRNA-), and DNA-based-vaccines.

Peptide vaccines are made up of small pieces of mutated KRAS protein. Pan *et al*^36^ have formulated a multi-peptide vaccine that targets different epitopes of *KRAS*, utilizing a longer peptide with an affinity for MHC class II rather than the shorter MHC class I restricted peptides. The vaccine demonstrated a 100 % sequence homology between humans and mice and successfully reduced surface tumors in the lungs of vaccinated mice. Another novel intranasal peptide vaccine is being developed to induce a local *KRAS*-specific immune response in the lungs through a mucosal approach.^121^

DNA plasmid vaccines are thought to be more effective than protein- or antigen-based vaccines. GI-4000 is a yeast-based recombinant DNA vaccine that expresses different *KRAS* mutations. It has undergone a phase 2 clinical trial where it was administered as a consolidation therapy to patients with stage I-III lung adenocarcinomas harboring *KRAS* G12C, G12D, or G12V mutations.^122^ Out of the 24 patients enrolled, 50 % were able to develop an immune response against mutant *KRAS*.^122^

Lastly, mRNA vaccines are similar to DNA vaccines, except they can induce an immune response without genetic modification. mRNA-5671/V941 targeting some of the most common *KRAS* mutations (G12D, G12V, G13D, and G12C) is undergoing phase 1 clinical trial.^123^

#### HapImmune antibodies

Cancer-specific antigens are also created from the FDA-approved *KRAS* G12C inhibitors, sotorasib and osimertinib, by using fragments of these inhibitors to develop hapten-peptides that form neoantigens.^124^ These neoantigens would then selectively mark cancer cells and enable engineered antibodies (HapImmune antibodies) to kill the inhibitor-resistant lung cancer cells specifically.^43^^,^^124^

## Conclusions

Despite the advancement in cancer treatments and checkpoint inhibitor immunotherapies, *KRAS* mutation-associated lung cancer remains the most challenging and difficult-to-treat cancer type with limited success. Primary and secondary resistance presents significant challenges in treating *KRAS*-mutated lung cancer despite the initial clinical success of *KRAS* G12C inhibitors. Due to the inevitable development of resistance, a single agent of the *KRAS* inhibitor is not likely efficacious in overcoming the heterogeneity of the *KRAS* mutant lung cancer. Although direct inhibition of the mutant *KRAS* may be the best therapeutic approach, therapies that target the mediators of the upstream and downstream RAS pathways could be combined with these direct *KRAS* inhibitors to overcome feedback activation. Currently, several combination approaches are being tested in the pre-clinical setting, with the most predominant approach being the use of RTK and SHP2/SOS inhibitors. Advances in therapeutic strategies, including developing new direct and indirect *KRAS* inhibitors, combination therapies, and immunological approaches, may offer promising solutions to overcome the inevitable resistance. Since binding pockets have yet to be discovered for the other *KRAS* isoform mutants, it is recommended that their direct inhibitors in development be used with other indirect forms of inhibition and immunotherapy. These emerging therapies, particularly when used in combination, hold the potential to improve treatment outcomes for patients with *KRAS*-mutated NSCLC, offering hope for better management of this complex and aggressive cancer.

## Availability of data and materials

The datasets generated and analyzed during the current study are available in the ClinicalTrials.gov repository, https://clinicaltrials.gov/, and the FDA repository, https://fda.gov/.

## CRediT authorship contribution statement

**Cynthia Hsin-Ya Chao:** Writing – review & editing, Writing – original draft, Data curation. **Yuanpu Peter Di:** Writing – review & editing, Writing – original draft, Supervision, Conceptualization.

## Declaration of competing interest

The authors declared that they have no known competing financial interests or personal relationships that could have appeared to influence the work reported in this paper.

## Appendix Supplementary materials

Image, application 1
