# Supplementary material for: Mechanisms and current advances in treating KRAS-mutated lung cancer
Source: Chin Med J Pulm Crit Care Med. 2025 Sep 16;3(3):149–63. doi: 10.1016/j.pccm.2025.08.001 (PMC12529572; doi:10.1016/j.pccm.2025.08.001)
Supplement: Supplementary file 1 [file mmc1.docx]

| **Supplementary Table 1: Timeline of advancements in *KRAS*-targeted therapies.** | | |
| --- | --- | --- |
| Year | Event | Ref. |
| 1967 | Kirsten sarcoma virus identified as a sarcoma inducing retrovirus | ^29^ |
| 1984 | *KRAS* identified as an oncogene in lung cancer | ^30^ |
| 2012 | MEK inhibitors used to block the RAF-MEK-ERK pathway | ^31^ |
| 2012 | PI3K/mTOR inhibitors used to target the PI3K–AKT pathway activated by *KRAS* mutation | ^32^ |
| 2013 | Allosteric binding pocket on *KRAS* G12C discovered | ^28^ |
| 2014 | DNA vaccine showed anti-tumor effect in mutant *KRAS* animal models | ^33^ |
| 2016 | Emergence of adoptive cell therapy targeting *KRAS* mutation | ^34^ |
| 2016 | Telomerase targeted due to upregulation by *KRA*S mutations | ^35^ |
| 2017 | Multi-peptide KRAS vaccine prevents *KRAS* mutant lung cancer in mice | ^36^ |
| 2019 | *KRAS* G12C inhibitors entered the clinical trial | ^37^ |
| 2020 | SHP2 inhibition is used to attenuate oncogenic signaling downstream of *KRAS* | ^38^ |
| 2020 | SOS1 inhibition is used to block *KRAS* activation | ^39^ |
| 2020 | First PROTAC (LC-2) developed to target *KRAS* | ^40^ |
| 2021 | U.S. FDA approved Sotorasib, the first *KRAS* G12C inhibitor | ^41^ |
| 2021 | Acquired resistance to *KRAS* G12C inhibitors reported | ^42^ |
| 2022 | HapImmune antibodies introduced | ^43^ |
| 2023 | Pan-KRAS inhibitor in the pipeline | ^44^ |
| 2024 | KRAS-ON inhibitors developed | ^45^ |
| 2024 | Pan-KRAS degrader emerged | ^46^ |

AKT: Protein kinase B; ERK: Extracellular signal-regulated kinase; FDA: U.S. Food and Drug Administration; G12C: Glycine-to-cysteine substitution at codon 12 of *KRAS*; *KRAS*: Kirsten rat sarcoma viral oncogene homolog; KRAS-ON inhibitors: Inhibitors targeting activated KRAS signaling pathways; LC-2: First PROTAC degrader targeting KRAS; MAPK: Mitogen-activated protein kinase; MEK: MAPK/ERK kinase; mTOR: Mammalian target of rapamycin; NSCLC: Non-small cell lung carcinoma; PI3K: Phosphoinositide 3-kinase; PI3K: Phosphoinositide 3-kinase–protein kinase B pathway; PROTAC: Proteolysis-targeting chimera; RAF: Rapidly accelerated fibrosarcoma; Ref.: Reference; SHP2: SH2 domain-containing phosphatase 2; SOS1: Son of sevenless homolog 1.
